# Maggot Extract Inhibits Cell Migration and Tumor Growth by Targeting HSP90AB1 in Ovarian Cancer

**DOI:** 10.3390/jcm11216271

**Published:** 2022-10-25

**Authors:** Daojuan Wang, Rong Wang, Mengru Cai, Yaling Zhang, Zhengquan Zhu, Yajing Weng, Lei Wang, Ying Huang, Ronghui Du, Xiaoke Wu, Gaojian Tao, Yong Wang

**Affiliations:** 1 The Affiliated Nanjing Drum Tower Hospital, Medical School, Nanjing University, Nanjing 210008, China; 2 State Key Laboratory of Analytical Chemistry for Life Science & Jiangsu Key Laboratory of Molecular Medicine, Medical School, Nanjing University, Nanjing 210093, China; 3 School of Medicine, Jiaxing University, Jiaxing 314001, China; 4 Department of Clinical Laboratory, Affiliated Hospital of Integrated Traditional Chinese and Western Medicine, Nanjing University of Chinese Medicine, Jiangsu Province Academy of Traditional Chinese Medicine, Nanjing 210028, China; 5 Department of Obstetrics and Gynecology, First Affiliated Hospital, Heilongjiang University of Chinese Medicine, Harbin 150040, China

**Keywords:** maggot extracts, ovarian cancer, cell proliferation, cancer metastasis, HSP90AB1

## Abstract

Ovarian cancer is one of the most lethal gynecological malignancies, because of metastatic dissemination with poor late clinical therapy. Maggots have been used in traditional Chinese medicine, where they are also known as ‘Wu Gu Chong’. Previous studies have indicated that maggot extract (ME) was beneficial for the treatment of gastric cancer when combined with other drugs, but the effect on anti-ovarian cancer and the underlying mechanism remains unclear. The aim of this study was to investigate the effects of ME on suppressing the proliferation and migration of ovarian cancer cells, and to clarify the underlying mechanism. In this research, Cell Counting Kit-8 (CCK-8), colony formation assay, and luciferase-positive cell quantification assay were employed to identify the inhibitory effects of ME on cell proliferation. Then, the pro-apoptosis and anti-metastasis effects of ME were explored by Western blot, dual annexin V-fluorescein isothiocyanate/propidium iodide (FITC/PI) assay, immunofluorescent staining, and wound-healing assay. We further established a xenograft model by subcutaneously or intraperitoneally injecting BALB/c nude mice with SKOV3 cells stably expressing luciferase, and the mice were treated with ME. The results showed that ME therapy effectively restrained the growth and metastasis of ovarian tumors in vivo. Furthermore, the mRNA levels of cancer factors including heat shock protein 90 alpha family class B member 1 (*HSP90AB1*), *MYC*, and insulin like growth factor 1 receptor (*IGF1R*) were analyzed by quantitative real-time PCR assay to explore the possible antitumor mechanisms of ME. Next, HSP90 ATPase activity was inhibited by geldanamycin in A2780, and the cell viability was shown to be dramatically reduced, decreasing further with the combination of ME and cisplatin. In turn, HSP90AB1 overexpression effectively inhibited the effect of ME in suppressing capability for cell viability and migration. In addition, HSP90AB1 overexpression limited the ability of ME to inhibit expression of MYC and IGF1R, while the opposite effect was observed for expression of pro-apoptosis protein caspase3 and BAX. Therefore, this study confirmed the potential roles and mechanisms of ME in inhibiting the growth and metastasis of ovarian tumors and promoting apoptosis of ovarian cancer cells by inhibiting overexpression of HSP90AB1.

## 1. Introduction

Ovarian cancer is one of the most lethal gynecologic malignancies, with a poor survival rate [1,2,3]. In recent years, the quantities of new cases and deaths from ovarian cancer have substantially increased worldwide [4]. According to statistics, in 2020, patients diagnosed with ovarian cancer in China accounted for approximately 17.6% of total global ovarian cancer cases, and the number of deaths caused by ovarian cancer accounted for approximately 18.1% of global total [5]. Extensive peritoneal dissemination at the time of diagnosis is a characteristic of ovarian cancer, and culminates predominantly in omental and bowel metastasis [6]. Epithelial ovarian cancer is the most common ovarian cancer, accounting for almost 90% of all ovarian cancer cases [7,8]. Many anticancer agents have been developed and clinically validated in platinum (Pt)-sensitive epithelial ovarian cancer patients, including those with high-grade serous ovarian cancer (HGSOC). However, most patients with HGSOC develop Pt-resistance, leading to almost inevitably fatal refractory disease, with a five-year survival rate of only 43% [2]. At present, chemoresistance and metastasis remain the major challenges for ovarian cancer treatment [2,9,10]. Therefore, understanding the underlying molecular mechanisms associated with the onset of Pt-resistance, and establishing a more accurate method of refining ovarian cancer treatment, are urgent unmet clinical needs. 

Heat shock protein 90 (HSP90) is a vital chaperone protein conserved across all organisms, which controls the stability and activity of chaperone proteins and maintains the functions of proteins in tumors [11,12,13,14]. Recent studies have illustrated that HSP90 is increased in colorectal cancer, non-small cell lung cancer, hepatocellular cancer, and breast cancer, causing poor survival rates [15,16,17]. HSP90 may promote tumorigenesis, in part because of its enhanced affinity for ATP and ATPase activity in cancer cells [18,19]. Snigireva et al. reported that HSP90 is related to cancer cell migration and invasion [20], but the role in ovarian cancer has not been fully studied. HSP90AA1 and HSP90AB1 are two isoforms of HSP90. Numerous studies have demonstrated that HSP90AA1 and HSP90AB1 participate in tumorigenesis, and their overexpression promotes angiogenesis, metastasis, and differentiation of cancer cells [18,21,22]. Here, we aimed to investigate whether traditional Chinese medicine was effective for suppressing cell proliferation and migration in ovarian cancer by targeting HSP90. 

Maggots are the larvae of Lucilia sericata, a species of blowfly found in China, Europe, and the Americas [23,24]. Previous studies have found that the extracts or secretions of maggots have advantages for the repair of inflammatory oxidative stress and fibrosis in colitis [24,25]. Bexfield et al. reported that extracts or secretions from maggots exhibited potent thermally stable protease-resistant antibacterial activity in vitro. Metabolites and compounds of low molecular weight in maggot extracts (ME) that exert antimicrobial effects have been identified and tested in vitro [26,27,28]. Increasing evidence suggests that ME plays a positive role in gastric cancer and coma when combined with other drugs [29]. However, the role of ME in improving ovarian cancer has not been explored. Therefore, we tested ME in epithelial ovarian cancer cells, demonstrating a strong antitumor effect on xenograft models in vitro and in vivo, as well as a synergistic antitumor effect when combined with cisplatin. Furthermore, this study elucidated the mechanisms underlying the therapeutic effects of ME on tumor cells’ migration and growth.

## 2. Materials & Methods

### 2.1. ME Preparation

Maggot powder was obtained from Jiangsu Key Laboratory of Molecular Medicine, Medical School, Nanjing University. Details of the preparation method for ME have been reported in published articles [24,25]. The ME samples used in this study were prepared from the same batch.

### 2.2. Ovarian Cancer Cells Culture

The human ovarian cancer cell line A2780 (RRID: CVCL_0134) was purchased from Shanghai Pituo Biological Technology Co., Ltd. (Shanghai, China). SKOV3 (RRID: CVCL_0532) was purchased from Solarbio Science & Technology Co., Ltd. (Solarbio, Beijing, China). A2780 and SKOV3 were separately cultured in RPMI-1640 medium (Gibco, Thermo Fisher Scientific, Waltham, MA, USA) and DMEM medium (Gibco), containing 10% FBS (Gibco), 100 U/mL of penicillin, and 100 U/mL of streptomycin (Gibco). 

### 2.3. Luciferase Reporter Assay

A2780 and SKOV3 cells were seeded in 6-well plates and infected with lentiviral particles (Ubi-MCS-firefly-luciferase-IRES-puromycin) (GeneChem, Shanghai, China) along with HitransG P, and further treated with puromycin to screen for cells that expressed luciferase. 

### 2.4. Lentiviral Vector Preparation and Infection

HSP90AB1 overexpression lentiviral Ubi-MCS-3FLAG-SV40-EGFP-IRES-puromycin for human use was purchased from GeneChem (Shanghai, China). A2780 cells were infected with lentiviral particles along with lentiviral vectors for overexpressing HSP90AB1. 

### 2.5. Cell Viability Assay

A2780 and SKOV3 cell lines were seeded in 96-well plates (1 × 10^4^ cells/well). Cell viability was measured using Cell Counting Kit-8 (CCK-8, Vazyme Biotech, Nanjing, China) according to the manufacturer’s instructions. 

### 2.6. Cell Colony Formation Assay

SKOV3 cells were seeded into 6 cm petri dish (100 cells/dish). After seeding for 7 days, the cells were incubated with various concentrations of ME (0, 4, 8 mg/mL, respectively). Another 7 days later, the colonies were subsequently fixed with 4% formaldehyde, stained with 10% Giemsa stain (Solarbio, Beijing, China), and imaged using an inverted microscope (Mshot, Guangzhou, China). Three independent experiments were conducted.

### 2.7. Wound-Healing Assay

A2780 and SKOV3 cells (1 × 10^5^ cells/well) were seeded into 12-well plates, and scraped with a 10 μL sterile pipette tip until the cells achieved 80% confluence. Then, the scratched cells were further cultured with 3.2 μg/mL cisplatin/ME (6, 8 mg/mL) for another 48 h. Images were captured under inverted microscope (Mshot, Guangzhou, China). The wound closure rate was analyzed by Image J. 

### 2.8. Annexin V-Fluorescein Isothiocyanate/Propidium Iodide (FITC/PI) Assay

A2780 were seeded in 24-well plates at a density of 1 × 10^5^ cells/well and treated with cisplatin/ME for 72 h. Dual annexin V-FITC/PI binding assays detected apoptotic cells (Beyotime, Shanghai, China). Then cells were photographed using an Olympus laser-scanning confocal microscope (FV3000). 

### 2.9. Flow Cytometry Analysis

A2780 and SKOV3 cells were seeded in 6-well plates at a density of 3 × 10^5^ cells/well, and treated with 3.2 μg/mL cisplatin/ME (6, 8 mg/mL) for 72 h. Then, A2780 and SKOV3 cells were collected in a flow tube and stained with dual annexin V-FITC/PI. The precise ratio of apoptotic cells was analyzed using a flow cytometer (BD FACS Calibur, BD Biosciences, Franklin Lakes, NJ, USA).

### 2.10. In Vivo Xenograft Model 

Female BALB/c nude mice (*n* = 12), aged 6 weeks, weighted 16–18 g, were obtained from Nanjing Junke Biotechnology Corporation. The mice were housed in a specific-pathogen-free (SPF) environment (Jiangsu Key Laboratory of Molecular Medicine), in a temperature- and humidity-controlled facility with 12-h light/dark cycles. 

All the mice were subcutaneously or intraperitoneally injected with 1 × 10^7^ SKOV3 cells (100 μL in saline), respectively. Then, mice were randomly divided into two groups (control, ME, *n* = 3 in each group). Seven days after injection, mice were anesthetized, intraperitoneally injected with D-luciferin (150 mg/kg body weight), imaging data were acquired at 10 min thereafter, and the data were analyzed using Living Image software (LB 983 NC100, Berthold Technologies GmbH & Co. KG, Bad Wildbad, Germany). Mice receiving ME treatment were administered with ME (1 g/kg, three times a week, oral) daily for four consecutive weeks. After 5 weeks of cell inoculation, imaging data were again acquired using Living Image software (LB 983 NC100, Germany). Then, the mice were euthanized in order to assess tumor load, and tumors were collected for molecular analysis.

### 2.11. Immunofluorescence (IF)

A2780 cells were seeded onto 12-well plates with round glass. Following treatment with cisplatin or MEs, the cells were fixed, permeabilized, and blocked. Next, cells were incubated with antibodies against p-p53 (Abcam, Cambridge, UK) overnight at 4 °C. Then, cells were incubated with fluorescent secondary antibodies, and counterstained with DAPI (Beyotime, Shanghai, China) at room temperature. Images were photographed using an Olympus laser-scanning confocal microscope (FV3000).

### 2.12. Western Blot

Cell lysates were generated by electrophoresis on 12% SDS-PAGE, and the proteins were transferred to polyvinylidene difluoride membranes (IPVH00010, Merck Millipore, Burlington, MA, USA), and then incubated with primary antibodies overnight at 4 °C. Next, the membranes were probed with the appropriate secondary antibodies for 1.5 h. The blots were visualized using chemiluminescent detection (Vazyme Biotech, Nanjing, China) and analyzed by Image J software. Loading was normalized with GAPDH. 

### 2.13. Quantitative Real-Time PCR (qRT-PCR)

Total RNA from cells was extracted using RNA Miniprep reagent (Beyotime, China) according to the instructions, and cDNA was synthesized with a reverse transcription kit (Vazyme Biotech, Nanjing, China). SYBR Green PCR Master Mix (Vazyme Biotech, Nanjing, China) was applied to analyze the relative expression of *MYC*, *HSP90AB1* and insulin like growth factor 1 receptor (*IGF1R*). qRT-PCR was performed with the ABI Viia 7 Real-Time PCR system (ABI USA, Los Angeles, CA, USA). β-actin was used as an internal control. The primers are shown in Table 1. The critical threshold cycle (Ct) value was determined for each reaction, which was transformed into relative quantification data using the 2^−∆∆Ct^ method.

### 2.14. Statistics

All statistical analyses were performed with GraphPad (Prism 7.00). Comparisons between two groups for statistical significance were assessed using a two-tail Student’s *t*-test. *p* value < 0.05 was considered statistically significant. All data presented are from at least three independent experiments.

## 3. Results

### 3.1. ME Suppresses Cell Proliferation in Ovarian Cancer

**Figure 1:**
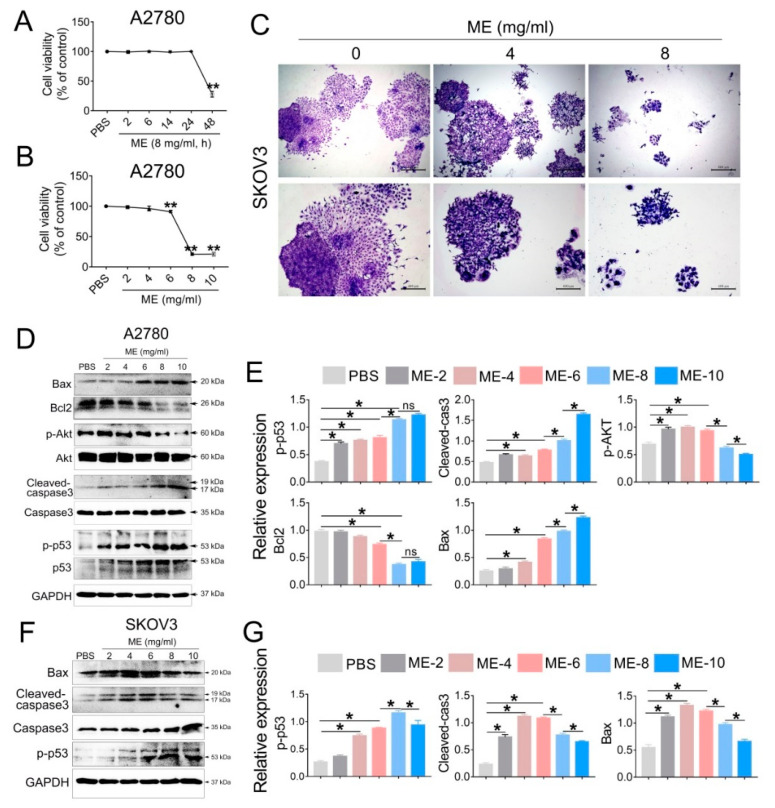
Maggot extract (ME) treatment inhibits cell proliferation in A2780 and SKOV3 cells. A2780 and SKOV3 cells were treated with different concentrations (0, 2, 4, 6, 8, 10 mg/mL) of ME for 48 h. (**A**) A2780 cells were treated with 8 mg/mL ME for 2, 6, 14, 24, 48 h. Cell viability was assayed using a CCK-8 kit. (**B**) A2780 cells were treated with different concentrations of ME for 48 h. Cell viability was assayed using a CCK-8 kit. (**C**) SKOV3 cells were seeded into 6 cm petri dishes and incubated with various concentrations of ME, respectively. The colonies were stained with 10% Giemsa stain and imaged using an inverted microscope. (**D**) The expression of apoptosis-related proteins in A2780 was analyzed by Western blot. (**E**) The panel on the right shows the quantitative analysis results. (**F**) The expression of apoptosis-related proteins in SKOV3 was analyzed by Western blot. (**G**) The panel on the right shows the quantitative analysis results. Three independent experiments were performed with similar results. Data are shown as mean ± SEM. ns, not significant, * *p* ≤ 0.05, ** *p* ≤ 0.01.

To investigate the antitumor effect of ME, A2780 cells were treated with 8 mg/mL ME, and the cell viability was analyzed at different time points (2, 6, 14, 24, 48 h). The results showed that cell viability was significantly decreased after ME treatment for 48 h (Figure 1A). We further treated A2780 cells with different concentrations of ME (2, 4, 6, 8, 10 mg/mL) for 48 h, and observed that the cell viability was downregulated approximately twenty percent with 6 mg/mL ME treatment. When the concentrations of ME reached 8 or 10 mg/mL, the cell viability was downregulated by about eighty percent (Figure 1B). Furthermore, colony-formation assay of SKOV3 cells was performed, revealing that, as expected, the clonogenic ability of SKOV3 cells was drastically decreased after incubation with 8 mg/mL ME (Figure 1C). Western blot analysis showed that the expression of pro-apoptotic proteins in A2780 cells, including p-p53, cleaved caspase3, and BAX, was dramatically increased with greater ME concentrations, and the expression of anti-apoptotic proteins, such as p-Akt and BCL2, was markedly reduced (Figure 1D,E). Similar results were observed in SKOV3 cells (Figure 1F,G).

### 3.2. ME Enhances the Antitumor Effect of Cisplatin in Ovarian Cancer

**Figure 2:**
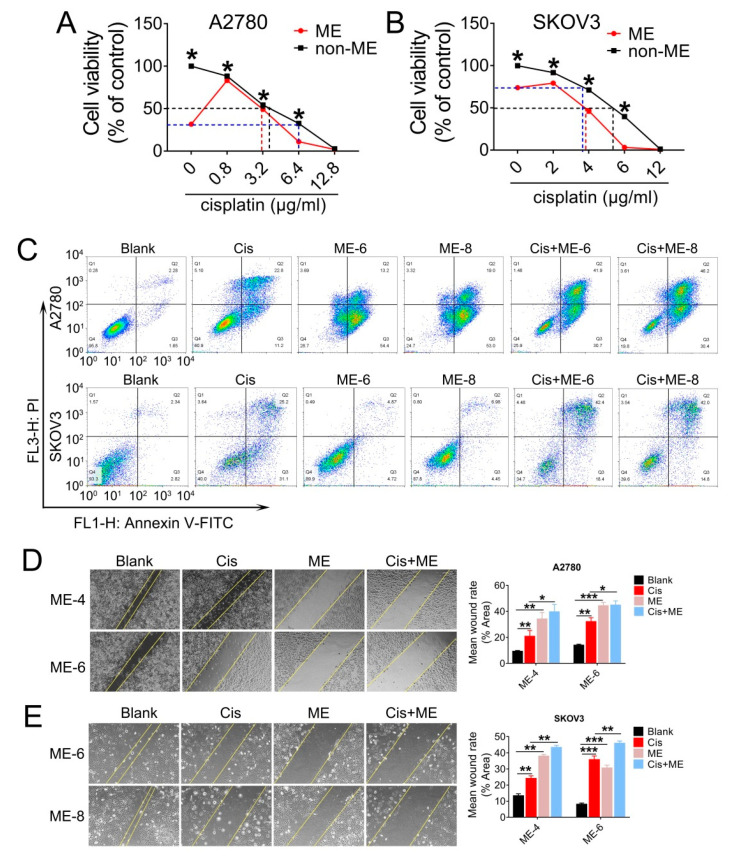
ME treatment promotes cell apoptosis and metastasis in ovarian cancer cells. A2780 and SKOV3 cells were treated for 48 h with different concentrations of cisplatin combined with 8 mg/mL ME. (**A**) Cell viability of A2780 was assayed using a CCK-8 kit. (**B**) Cell viability of SKOV3 was assayed using a CCK-8 kit. (**C**) A2780 and SKOV3 were double-stained with dual annexin V-fluorescein isothiocyanate/propidium iodide (FITC/PI), then analyzed by flow cytometric assay. (**D**) Wound-healing assays were performed for A2780, the quantification of wound-closure rates is shown on the right. (**E**) Wound-healing assays were performed for SKOV3, and the quantification of wound-closure rates is shown on the right. Three independent experiments were performed with similar results. Data are shown as mean ± SEM. * *p* ≤ 0.05, ** *p* ≤ 0.01, *** *p* ≤ 0.001.

Having observed a striking inhibitory effect of ME on ovarian cancer cell growth, we decided to further investigate whether ME enhanced the antitumor effect of cisplatin in ovarian cancer. A2780 and SKOV3 cells were treated with various concentrations of cisplatin combined with 8 mg/mL ME. The results showed that the cell viability of A2780 and SKOV3 with 8 mg/mL ME treatment was equivalent to viability after 6.4 and 3.5 μg/mL cisplatin treatment, respectively (Figure 2A,B). Furthermore, we found that about 4 μg/mL cisplatin was required for fifty percent cell viability in A2780. When combined with ME treatment, the concentration of cisplatin was reduced to 3 μg/mL (Figure 2A). Similarly, about 5.3 μg/mL cisplatin was required for fifty percent cell viability in SKOV3, and the concentration was reduced to 3.8 μg/mL when combined with ME treatment (Figure 2B). We analyzed cell apoptosis rates using Annexin V/PI assay. Flow cytometric analysis of A2780 and SKOV3 cells with cisplatin treatment revealed a tenfold increase in late apoptosis rate compared with the blank group, while the apoptosis rate of ME combined with cisplatin treatment was twenty times higher than the blank group (Figure 2C). The results detected by confocal fluorescence microscopy were consistent with the data described above (Supplementary Figure S1). To further examine whether ME can repress the metastasis of ovarian cancer cells, we performed wound-healing assays for the A2780 and SKOV3 cells. Our data showed that cisplatin can inhibit the migration of A2780 and SKOV3 cells to a limited extent. However, ME treatment alone or combined with cisplatin more markedly suppressed the migration of A2780 and SKOV3 cells (Figure 2D,E). These results suggest that ME therapy can effectively enhance the antitumor effect of cisplatin in ovarian cancer. 

### 3.3. ME/Cisplatin Combination Therapy Promotes Cell Apoptosis in Ovarian Cancer

**Figure 3:**
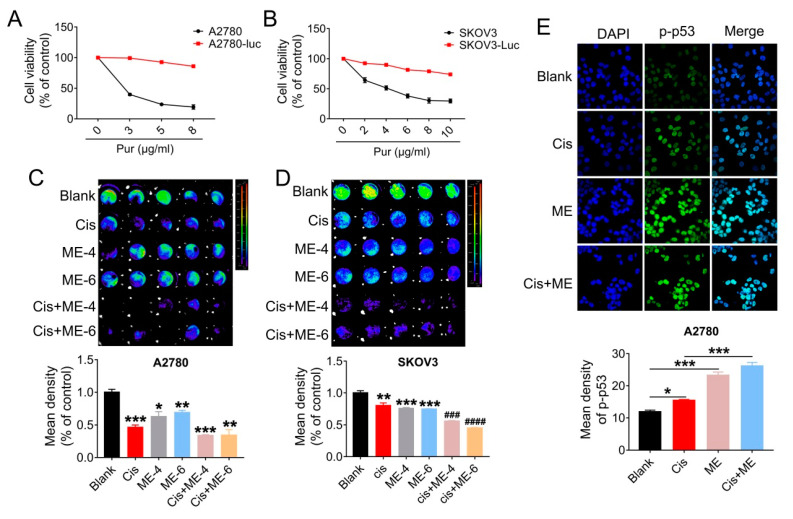
ME/cisplatin combination therapy promotes cell apoptosis in ovarian cancer. A2780 and SKOV3 cells were infected with lentiviral particles to express luciferase, and then treated with MEs/cisplatin. (**A**) A2780 and (**B**) SKOV3 cells were treated with different concentrations of puromycin to verify the transfection efficiency of luciferase. Cell viability was assayed using a CCK-8 kit. (**C**) Luciferase-positive A2780 and (**D**) SKOV3 were analyzed using Living Image software and a GloMax^®^ 96-microplate luminometer. The lower panels show the results of the quantitative analysis. * *p* ≤ 0.05, ** *p* ≤ 0.01, *** *p* ≤ 0.001 vs. blank; ^###^ *p* ≤ 0.001, ^####^ *p* ≤ 0.0001 vs. cis. (**E**) Levels of p-p53 in A2780 cells were measured by immunofluorescence staining (400×). The lower panel shows the results of the quantitative analysis of p-p53 in A2780. * *p* ≤ 0.05, *** *p* ≤ 0.001. Three independent experiments were performed with similar results. Data are shown as mean ± SEM.

To further confirm that ME can enhance the antitumor effect of cisplatin in ovarian cancer, cells were transfected with luciferase-expressing lentivirus, and analyzed using Living Image software (PerkinElmer Company, Hopkinton, MA, USA) and a GloMax^®^ 96-microplate luminometer (Promega Corp., Madison, WI, USA). We first screened for luciferase expression in A2780 and SKOV3 cells, using puromycin (Figure 3A,B). Then, the luciferase-positive ovarian cancer cells were quantified. The results showed that cell viability in A2780 and SKOV3 cells following ME/cisplatin combination therapy was markedly decreased, to a level dramatically lower than cisplatin treatment alone (Figure 3C,D). We subsequently analyzed the expression of p-p53 in A2780 cells using immunofluorescent staining. We found that ME combined with cisplatin increased the expression of p-p53 in A2780 cells, to a level markedly higher than cisplatin treatment alone (Figure 3E). Taken together, these data indicate that ME combined with cisplatin can more effectively promote cell apoptosis in ovarian cancer.

### 3.4. ME Treatment Inhibits the Growth of Ovarian Tumor In Vivo

**Figure 4:**
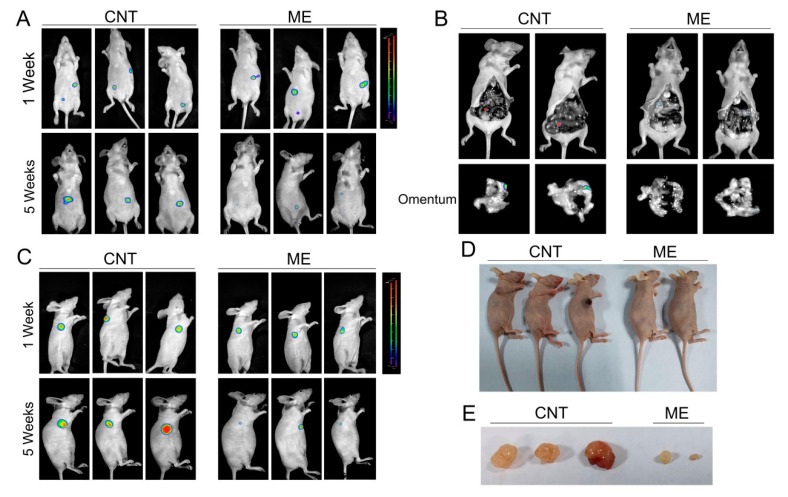
ME treatment inhibits the growth of ovarian tumors in vivo. (**A**) Nude mice were implanted with SKOV3 by two methods, i.e., subcutaneous injection and intraperitoneal injection. The two kinds of tumor-bearing mice were divided into two groups (*n* = 3), respectively. Mice receiving ME treatments were administered with ME (1 g/kg, three times a week, oral) daily for four consecutive weeks. After 1 and 5 weeks of cell inoculation, mice were anesthetized. (**A**) Intraperitoneal tumor-bearing mice were injected intraperitoneally with D-luciferin (150 mg/kg body weight); imaging data were acquired 10 min thereafter, and analyzed using Living Image software. (**B**) Intraperitoneal tumor-bearing mice were dissected. Imaging data of the omenta were acquired and analyzed using Living Image software. (**C**) Mice with subcutaneous tumors were injected intraperitoneally with D-luciferin and analyzed using Living Image software. (**D**) Pictures of mice with subcutaneous tumors. (**E**) Photographs of the morphologies of the tumors from each treatment group.

We further established a xenograft model by subcutaneous or intraperitoneal injection of SKOV3 cells, which stably expressed luciferase, in BALB/c nude mice, and treated the mice with ME. Tumors in the mice were detected using bioluminescence imaging. One week after cell inoculation, tumors were analyzed by bioluminescence imaging, and then the treatment groups of mice were intragastrically administered with ME. By 5 weeks after treatment with ME, the luciferase-positive areas had obviously decreased (Figure 4A,C). Intraperitoneal-injected tumors predominantly colonized in the intestine and omentum, and ME therapy could effectively suppress tumor metastasis to the omentum (Figure 4B). In addition, the sizes of subcutaneous tumors significantly decreased after ME treatment (Figure 4D,E). These findings indicate that ME treatment can inhibit the growth of tumors and metastasis in ovarian cancer.

### 3.5. ME Treatment Resists Tumors through Inhibiting the HSP90AB1/IGF1R/MYC Pathway in Ovarian Cancer 

**Figure 5:**
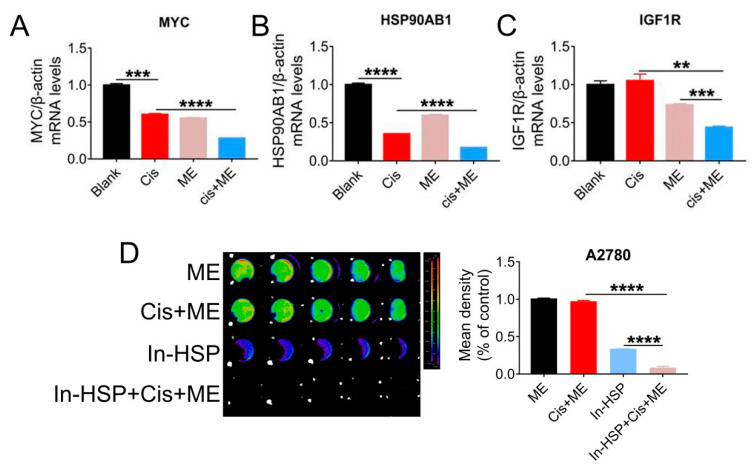
HSP90 ATPase inhibition with geldanamycin enhances the pro-apoptosis effect of ME in ovarian cancer cells. SKOV3 cells were treated with 3.2 μg/mL cisplatin and 6 mg/mL ME for 48 h. The mRNA levels of (**A**) MYC, (**B**) HSP90AB1, and (**C**) IGF1R were measured by real-time PCR. (**D**) The luciferase positive A2780 were analyzed using Living Image software and a GloMax^®^ 96-microplate luminometer. The quantification of luciferase-positive cells is indicated on the right. Three independent experiments were performed with similar results. Data are shown as mean ± SEM. ** *p* ≤ 0.01, *** *p* ≤ 0.001, **** *p* ≤ 0.0001.

To further explore the mechanisms by which ME enhances the antitumor effect of cisplatin, qRT-PCR assay was carried out to examine the expression of cancer pathway factors. We treated cells with cisplatin or ME or both, and the expression of cancer pathway factors including *MYC1*, *HSP90AB1*, and *IGF1R* was assessed. The mRNA levels of *MYC*, *HSP90AB1*, and *IGF1R* in SKOV3 cells were markedly decreased by ME treatment, and ME treatment combined with cisplatin further reduced the mRNA levels of *MYC*, *HSP90AB1*, and *IGF1R* (Figure 5A–C). To further confirm that ME can enhance cisplatin sensitivity by targeting HSP90, cells expressing luciferase were pretreated with geldanamycin, an inhibitor of HSP90 ATPase, followed by cisplatin and ME treatments. After inhibition of HSP90 ATPase activity in A2780 cells by geldanamycin, cell viability was dramatically reduced, and further decreased with ME/cisplatin combination therapy (Figure 5D). 

**Figure 6:**
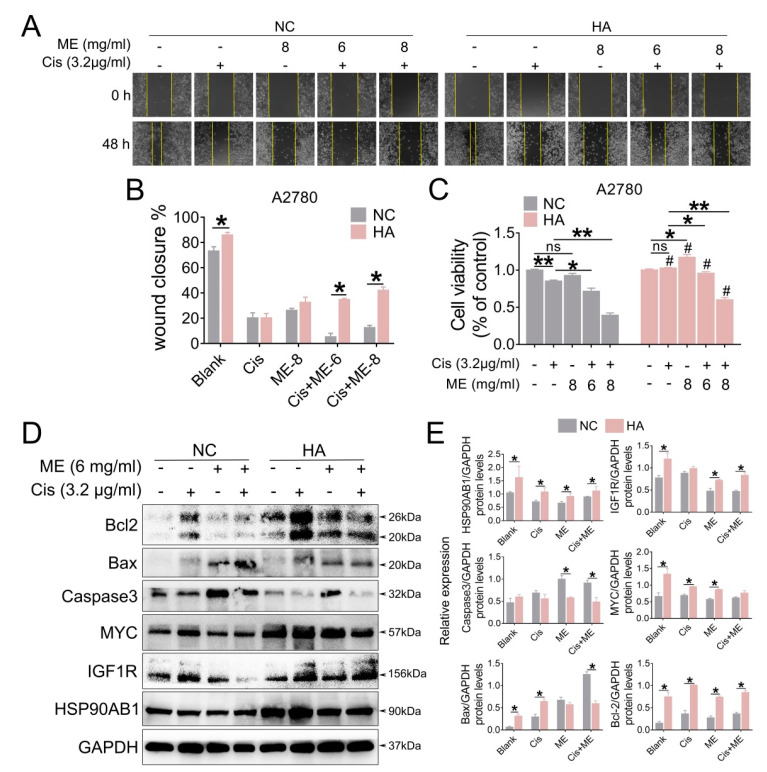
HSP90AB1 overexpression inhibits ME-induced metastasis and apoptosis in A2780 cells. A2780 cells were infected for 72 h with lentiviral particles marked with GFP to overexpress HSP90AB1. Then, cells were treated with cisplatin and ME. (**A**) Wound-healing assays were performed for the A2780 caells. (**B**) The panel shows the results of the quantitative analysis. (**C**) Cell viability of A2780 was assayed using a CCK-8 kit. # means HA vs. NC. (**D**) The protein expression of HSP90AB1, IGF1R, MYC, Caspase3, BAX, and BCL2 was analyzed by Western blot assay. (**E**) Panels indicate the results of quantitative analysis. Three independent experiments were performed with similar results. Data are shown as mean ± SEM. ns, not significant, * *p* ≤ 0.05, ** *p* ≤ 0.01; # *p* ≤ 0.05.

To gain a better understand of the molecular mechanism of ME in ovarian cancer, we overexpressed HSP90AB1 in A2780 cells by using lentivirus. To confirm the effect of HSP90AB1 on cell migration, we performed wound-healing assays for A2780 cells with overexpressed HSP90AB1. Our data showed that HSP90AB1 overexpression promoted the migration of A2780 cells. Moreover, HSP90AB1 overexpression hindered the limiting effect of ME on the migration of A2780 cells (Figure 6A,B). Cell viability was analyzed using CCK-8. The results showed that the cell viability markedly decreased with ME/cisplatin treatment before overexpression of HSP90AB1. After overexpression of HSP90AB1, cell viability with ME or cisplatin treatment alone or in combination was significantly higher than that of the negative control group (Figure 6C). Meanwhile, the protein expression of IGF1R, MYC, and BCL2 was markedly increased. Furthermore, HSP90AB1 overexpression inhibited not only the inhibitory effect of ME on IGF1R, MYC, and BCL2 in A2780 cells, but also the expansionary effect of ME on the expression of pro-apoptotic proteins caspase3 and BAX (Figure 6D,E). These data suggest that ME treatment resists ovarian cancer by inhibiting cancer factors HSP90AB1, IGF1R, and MYC.

## 4. Discussion

In the literature, HSP90AB1 overexpression has been identified as promoting the growth and metastasis of ovarian tumors. Our study found that ME treatment effectively reversed HSP90AB1 overexpression, downregulated the expression of cancer-related factors IGF1R and MYC, and exerted anti-tumorigenic effects in ovarian cancer. The ME used in this study was a soluble mixture from maggot larvae, which has been widely reported to play important roles in anti-inflammatory, antibacterial, and antioxidative stress, and even to have anti-cancer properties [24,25,29,30,31]. However, the molecular mechanism of ME in many therapeutic biological processes remains poorly understood. The underlying regulatory mechanism of ME on HSP90AB1 is of great significance for improving the clinical therapy of ovarian cancer. 

Because of its various biological activities, ME is currently approved by the Food and Drug administration for clinical use [32]. Our previous studies examined the potential toxicity of ME in vivo and in vitro, and no obvious clinical signs of toxicity were found in mice exposed to ME [24,25]. ME has mainly been used in the treatment of tissue necrosis and wound infection, with a few reports about its role in treating gastric cancer when combined with other drugs [29,33]. In this work, we demonstrated that ME is beneficial for promoting apoptosis and inhibiting migration of ovarian cancer in vivo or in vitro, even without cisplatin. 

Cisplatin and carboplatin are common chemotherapy drugs for ovarian cancer therapy, which exert antitumor effects by binding to DNA and forming cross-links, thus disrupting DNA structure and causing cell apoptosis [34,35]. The response of cells to DNA damage is a crucial determinant development for cancer therapy. However, dysregulation of chemotherapy drugs during that process may cause platinum resistance, and also systemic multiorgan failure. Thus, it is very important to find new drugs to enhance the sensitivity of ovarian cancer to cisplatin, and to reduce the use of chemotherapy drugs.

HSP90 is a molecular chaperone, involved in assisting signal transduction of cancer cells. HSP90 is considered a potential target for cancer therapy [11,36]. In addition, HSP90 has been reported to be overexpressed in Pt-resistant cancer cells, suggesting its potential role in the resistance mechanism. HSP90 inhibition in combination with cisplatin has a synergistic antitumor effect in drug-resistant epithelial ovarian cancer cells [37]. HSP90AB1 is one subtype of the HSP90 family, which is related to ATPase activity in cancer cells, and part of its function is to promote cell growth, metastases, invasion, and immune response [38,39,40]. Our findings confirmed that HSP90AB1 overexpression could enhance the tolerance of ovarian cancer cells to chemotherapeutic agents by activating drug-resistant proteins IGF1R and MYC, affecting the prognosis of patients. 

In the present study, A2780 cells were not strongly tumorigenic in mice, and SKOV3 cells stably expressing luciferase were utilized to establish xenograft models by subcutaneous or intraperitoneal injection in BALB/c nude mice, and mice were further treated with ME. Tumor size and metastases were markedly suppressed by ME treatment. Among the tested samples, the tumor of one subcutaneous tumor-bearing mouse treated with ME could hardly be seen, so we did not show this mouse in Figure 4D,E. HSP90AB1 overexpression mechanistically activates the proteins IGF1R and MYC, which nullifies the antitumor effect of ME. In turn, inhibition of HSP90AB1 further promotes ME-induced ovarian cancer cell apoptosis. Previous studies have consistently demonstrated that HSP90 as a druggable target reverses Pt resistance in ovarian cancer [11,37]. In addition, knockdown of HSP90 significantly inhibits cell proliferation in H08910 ovarian cancer cells [41]. Interestingly, while studying the mechanism of bone metastasis in breast cancer, Sun et al. found that HSP90AB1 functioned as a tumor suppressor in the extracellular domain and a tumor promoter in the intracellular domain [42,43]. These data suggest that HSP90AB1 might be a valuable new prognostic marker for ovarian cancer. Future studies might reveal whether ME could sensitize ovarian cancer cells towards platinum-based therapy by targeting HSP90. Meanwhile, analyzing the active agents of ME is important for developing specific drugs for ovarian cancer patients.

## 5. Conclusions

This study investigated the potential role of ME in inhibiting ovarian cancer growth and metastasis. Our data suggest that ME could suppress oncogenes IGF1R and MYC by inhibiting HSP90AB1 expression, further promoting apoptosis of ovarian cancer cells. Our study provides strong evidence that strategies for HSP90AB1 repression by combination of ME and cisplatin possess therapeutic potential in ovarian cancer.

## Figures and Tables

**Table 1:** Primers of the human genes used in the study.

Genes	Forward	Reverse
*β-Actin*	5′-AGCGAGCATCCCCCAAAGTT-3′	5′-GGGCACGAAGGCTCATCATT-3′
*MYC*	5′-CCTGGTGCTCCATGAGGAGAC-3′	5′-CAGACTCTGACCTTTTGCCAGG-3′
*HSP90AB1*	5′-CTCTGTCAGAGTATGTTTCTCGC-3′	5′-GTTTCCGCACTCGCTCCACAAA-3′
*IGF1R*	5′-CCTGCACAACTCCATCTTCGTG-3′	5′-CGGTGATGTTGTAGGTGTCTGC-3′

## Data Availability

The data that support the findings of this study are available from the corresponding author upon reasonable request.

## Supplementary Materials

The following supporting information can be downloaded at: https://www.mdpi.com/article/10.3390/jcm11216271/s1, Figure S1: ME treatment promotes apoptosis in A2780 cells. A2780 cells were treated with cisplatin (3.2 μg/mL) and ME (6 mg/mL) for 48 h. A2780 cells were double stained with Annexin V-FITC and PI. The cells were imaged for apoptosis detection using a FV3000 Olympus microscope.
