# Supplementary material for: Maggot Extract Inhibits Cell Migration and Tumor Growth by Targeting HSP90AB1 in Ovarian Cancer
Source: J Clin Med. 2022 Oct 25;11(21):6271. doi: 10.3390/jcm11216271 (PMC9657850; doi:10.3390/jcm11216271)
Supplement: Supplementary file 1 [file jcm-11-06271-s001.zip › jcm-1894822-supplementary.pdf]

Journal of
Clinical Medicine

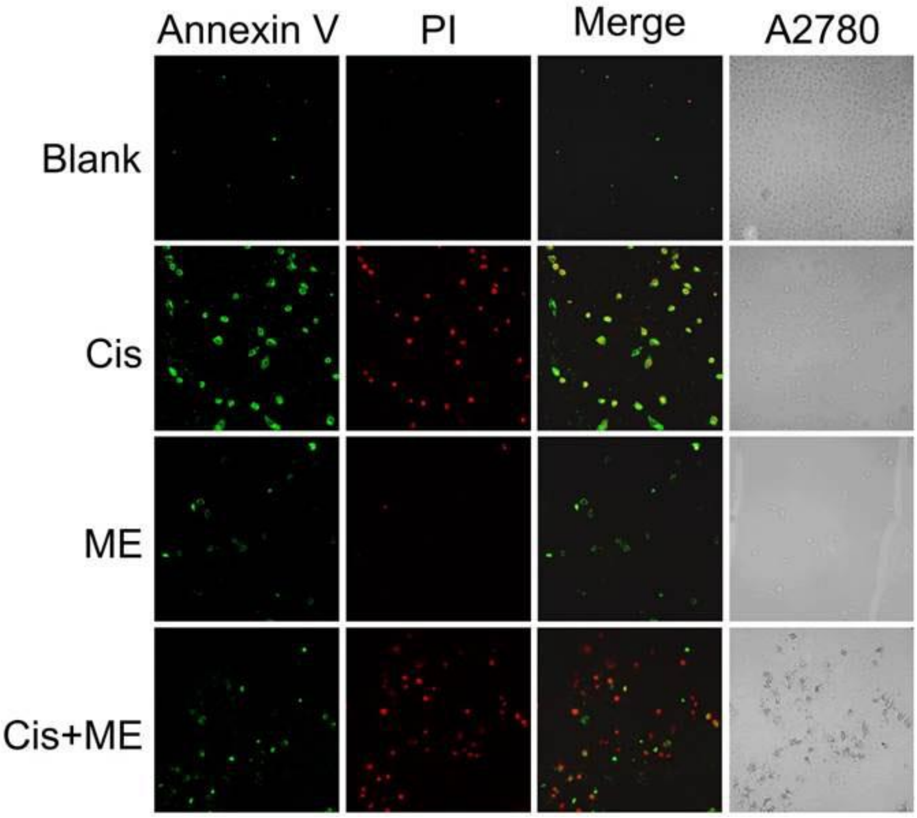

Figure S1. ME combined with cisplatin treatments promote apoptosis in A2780 cells. A2780 were treated with 3.2 μg/ml cisplatin and 6 mg/ml MEs for 48 h. A2780 cells were incubated with Annexin V-FITC and PI. The cells were imaged for late apoptosis detection using a FV3000 Olympus microscope.
